# Impact of cesarean section delivery and breastfeeding on infant gut microbiota at one year of age

**DOI:** 10.1186/1710-1492-10-S1-A24

**Published:** 2014-03-03

**Authors:** Meghan B Azad, Theodore Konya, David S Guttman, Catherine J Field, Radha S Chari, Malcolm R Sears, Allan B Becker, James A Scott, Anita L Kozyrskyj

**Affiliations:** 1 Pediatrics, University of Alberta, Edmonton, Alberta, Canada; 2 Dalla Lana School of Public Health, University of Toronto, Toronto, Ontario, Canada; 3 Cell & Systems Biology, University of Toronto, Toronto, Ontario, Canada; 4 Agriculture, Food & Nutritional Sciences, University of Alberta, Edmonton, Alberta, Canada; 5 Obstetrics & Gynecology, University of Alberta, Edmonton, Alberta, Canada; 6 Department of Medicine, McMaster University, Hamilton, Ontario, Canada; 7 Pediatrics & Child Health, University of Manitoba, Winnipeg, Manitoba, Canada; 8 Canadian Healthy Infant Longitudinal Development Study, Canada

## Background

The gut microbiota is essential to human health, playing central roles in host metabolism and immunity. In a pilot study, we previously demonstrated that mode of delivery and breastfeeding influence the infant gut microbiota at 4 months of age. Here, we examine the impact of these critical early-life exposures in a larger cohort at 12 months of age.

## Methods

The study comprised a sub-sample of 190 healthy term infants from one centre participating in the Canadian Healthy Infant Longitudinal Development (CHILD) birth cohort study. Mode of delivery was determined from hospital records, and mothers reported infant diet at 3, 6 and 12 months postpartum. Antibiotic exposure was documented from hospital and prescription records. Gut microbiota was characterized by Illumina 16S rRNA sequencing of fecal samples collected at 12 months.

## Results

Delivery and breastfeeding practices significantly influenced infant gut microbiota composition and diversity at one year of age. Regardless of breastfeeding or antibiotic exposure, infants delivered by emergency cesarean section had higher gut microbiota diversity compared to infants delivered vaginally or by elective cesarean section (p<0.001). Consistent with our pilot results at 4 months, emergency cesarean delivery was associated with lower relative abundance of *Bacteroides* (p<0.001); this difference was attenuated in breastfed infants. Independent of delivery mode or antibiotic exposure, and contrary to our findings at 4 months, breastfeeding exclusivity (none, partial or full) and duration (never, < 6 months, ≥ 6 months) were associated with progressively higher diversity, and increasing relative abundance of *Bifidobacteria* (p for trends all <0.01). Other taxa influenced by mode of delivery and breastfeeding included the Family Lachnospiraceae (increased following emergency cesarean delivery) and the Genera *Veillonella*, *Lactobacillus* and *Megasphera* (all increased among breastfed infants).

## Conclusions

Mode of delivery and breastfeeding are strong determinants of the infant gut microbiota, with persistent and potentially interactive effects throughout the first year of life. Breastfeeding appears to influence microbiota diversity differently at 4 versus 12 months of age, indicating that measures of diversity require cautious interpretation (including consideration of age at assessment), and that a single measure of nutrition may not adequately reflect the diverse exposures that occur during the weaning period. Ongoing research in the CHILD study will address the cumulative and long-term impact of these and other early-life exposures, and associated changes to the gut microbiota, on the development of allergic disease and other health outcomes.

